# Multiomics Identification of Potential Targets for Alzheimer Disease and Antrocin as a Therapeutic Candidate

**DOI:** 10.3390/pharmaceutics13101555

**Published:** 2021-09-24

**Authors:** Alexander T. H. Wu, Bashir Lawal, Li Wei, Ya-Ting Wen, David T. W. Tzeng, Wen-Cheng Lo

**Affiliations:** 1 The Ph.D. Program of Translational Medicine, College of Medical Science and Technology, Taipei Medical University, Taipei 11031, Taiwan; chaw1211@tmu.edu.tw; 2 Clinical Research Center, Taipei Medical University Hospital, Taipei Medical University, Taipei 11031, Taiwan; 3 TMU Research Center of Cancer Translational Medicine, Taipei Medical University, Taipei 11031, Taiwan; 4 Graduate Institute of Medical Sciences, National Defense Medical Center, Taipei 11490, Taiwan; 5 PhD Program for Cancer Molecular Biology and Drug Discovery, College of Medical Science and Technology, Taipei Medical University and Academia Sinica, Taipei 11031, Taiwan; d621108004@tmu.edu.tw; 6 Graduate Institute for Cancer Biology & Drug Discovery, College of Medical Science and Technology, Taipei Medical University, Taipei 11031, Taiwan; 7 Graduate Institute of Injury Prevention and Control, College of Public Health, Taipei Medical University, Taipei 11031, Taiwan; nsweili@gmail.com; 8 Department of Neurosurgery, Taipei Medical University-Wan Fang Hospital, Taipei 11031, Taiwan; 98142@w.tmu.edu.tw; 9 School of Life Sciences, The Chinese University of Hong Kong, Hong Kong 999077, China; allqwdd@gmail.com; 10 School of Medicine, College of Medicine, Taipei Medical University, Taipei 11031, Taiwan; 11 Department of Neurosurgery, Taipei Medical University Hospital, Taipei 11031, Taiwan

**Keywords:** Alzheimer’s disease, biomarker identification, in silico pharmacological analyses, antrocin, therapeutic innovation

## Abstract

Alzheimer’s disease (AD) is the most frequent cause of neurodegenerative dementia and affects nearly 50 million people worldwide. Early stage diagnosis of AD is challenging, and there is presently no effective treatment for AD. The specific genetic alterations and pathological mechanisms of the development and progression of dementia remain poorly understood. Therefore, identifying essential genes and molecular pathways that are associated with this disease’s pathogenesis will help uncover potential treatments. In an attempt to achieve a more comprehensive understanding of the molecular pathogenesis of AD, we integrated the differentially expressed genes (DEGs) from six microarray datasets of AD patients and controls. We identified ATPase H+ transporting V1 subunit A (*ATP6V1A*), BCL2 interacting protein 3 (*BNIP3*), calmodulin-dependent protein kinase IV (*CAMK4*), TOR signaling pathway regulator-like (TIPRL), and the translocase of outer mitochondrial membrane 70 (*TOMM70*) as upregulated DEGs common to the five datasets. Our analyses revealed that these genes exhibited brain-specific gene co-expression clustering with *OPA1*, *ITFG1*, *OXCT1*, *ATP2A2*, *MAPK1*, *CDK14*, *MAP2K4*, *YWHAB*, *PARK2*, *CMAS*, *HSPA12A*, and *RGS17*. Taking the mean relative expression levels of this geneset in different brain regions into account, we found that the frontal cortex (BA9) exhibited significantly (*p* < 0.05) higher expression levels of these DEGs, while the hippocampus exhibited the lowest levels. These DEGs are associated with mitochondrial dysfunction, inflammation processes, and various pathways involved in the pathogenesis of AD. Finally, our blood–brain barrier (BBB) predictions using the support vector machine (SVM) and LiCABEDS algorithm and molecular docking analysis suggested that antrocin is permeable to the BBB and exhibits robust ligand–receptor interactions with high binding affinities to CAMK4, TOMM70, and T1PRL. Our results also revealed good predictions for ADMET properties, drug-likeness, adherence to Lipinskís rules, and no alerts for pan-assay interference compounds (PAINS) Conclusions: These results suggest a new molecular signature for AD parthenogenesis and antrocin as a potential therapeutic agent. Further investigation is warranted.

## 1. Introduction

Alzheimer’s disease (AD), which is characterized by functional impairment, progressive cognitive dysfunction, and memory loss, is the most frequent cause of neurodegenerative dementia in aging populations, affecting nearly 50 million people worldwide [1]. Worldwide prevalence rates range from 1.0% at 60 years of age to 30–50% by 85 years of age [2,3]. The etiology of AD is multifactorial and involves complex interactions between genetic, lifestyle, and environmental factors; however, about 70% of the risk is believed to be genetic [4].

The pathology of AD is irreversible, and early stage diagnosis is paramount to halting the progression of the disease and avoiding deterioration [5,6]. Major clinical manifestations of AD and other dementias are mild cognitive impairment (MCI) and subjective cognitive decline (SCD). However, the early detection of AD, SCD, MCI, and other dementias is still challenging [7,8]. The current diagnostic tools have several limitations and are unable to detect the disease in its early stages [9].

In addition, there is currently no effective treatment for AD, and presently available drugs do not modify the disease because they are unable to halt its progression [10]. The specific genetic alterations and pathological mechanisms of the development and progression of dementia remain poorly understood [11], and research in identifying more-important genes and molecular pathways associated with AD pathogenesis will help uncover potential treatments.

Gene expression microarrays are widely used to comprehensively measure the genome-wide expression profiles of clinical samples, aiding in the identification of disease-related genes [12]. Previous studies have investigated gene expression changes in the brain tissues of patients with AD and other neurodegenerative dementias [13,14,15,16,17,18]. The public availability of the results from those studies offers the possibility of analyzing the transcriptomic data to identify potential drug targets and to develop appropriate therapeutic strategies.

Natural products, particularly medicinal plants, represent the sources of the lead compounds for therapeutic development [19,20]. They are perceived as safer and offer fewer chances of drug resistance than most conventional therapies, which are limited by their undesirable side effects and drug resistance. *Antrodia cinnamomea* (AC) is a unique fungal species that is found exclusively in Taiwan and is traditionally used by indigenous herbalists to treat various diseases [21]. Among the many phytochemicals identified in AC, antrocin is a sesquiterpene lactone with established anti-neoplastic and immune-modulating functions [22]. Our previous data indicated that antrocin suppresses several biological signaling pathways, including the focal adhesion kinase (FAK)/paxillin phosphatidylinositol 3-kinase (PI3K)/Akt/mitogen-activated protein kinase (MAPK), extracellular signal-regulated kinase (ERK)/c-Fos/matrix metalloproteinase (MMP)-2, Akt/mammalian target of rapamycin (mTOR)/glycogen synthase kinase (GSK)-3β/nuclear factor (NF)-κB, Janus kinase (JAK)/signal transduction and activator of transcription (STAT3), β-catenin/Notch1/Akt, and insulin-like growth factor-1 receptor (IGF-1R) [23,24,25,26,27]. In addition, the activation of apoptotic markers including Fas, DR5, Bax, caspase–3, –8, and –9 were also implicated in the pharmacological activities of antrocin; however, whether antrocin can modulate the gene alterations associated with AD remains unclear.

**Figure 1:**
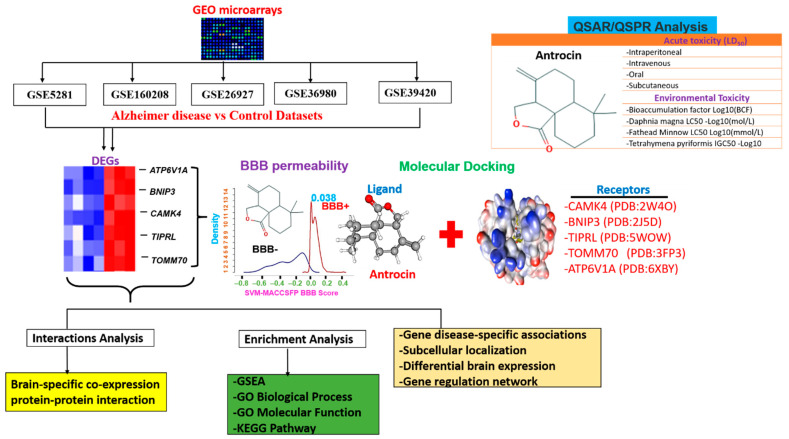
The flow chart of the study design for in silico identification of potential targets for Alzheimer’s disease and antrocin as a therapeutic candidate.

We analyzed five AD microarray datasets and identified *ATP6V1A*, *BNIP3*, *CAMK4*, *TIPRL,* and *TOMM70* as upregulated DEGs that are common in all five datasets (Figure 1). These genes covered a wide range of biological pathways related to brain function and disabilities. Furthermore, our in silico blood–brain barrier (BBB) permeability and molecular docking analysis suggested that antrocin is permeable to the BBB and exhibited robust ligand–receptor interactions with high binding affinities to CAMK4, TOMM70, and T1PRL, suggesting its potential as a new therapeutic agent for AD.

## 2. Methods

### 2.1. Collection of Microarray Data of Early Onset Alzheimer’s Disease (AD)

The microarray AD datasets were collected from the NCBI GEO, a public functional genomics data repository of high-throughput gene expression data (http://www.ncbi.nlm.nih.gov/geo/, accessed on 21 September 2021). The five datasets that we used are described in Table 1. The GSE26927 (GPL6255 (Illumina humanRef-8 v2.0 expression beadchip) dataset (released on Jan. 29, 2011) consists of the microarray data from postmortem central nervous system (CNS) tissues of 63 AD patients and 54 control samples [13]. The GSE160208 (GPL29311, NanoString nCounter Human Myeloid Innate Immunity Panel v2) dataset (released Jan. 8, 2021) consists of gene expression data from the frontal cortex (FC) and cerebellum (CB) of 27 Creutzfeldt–Jakob diseases (CJD) patients and 20 normal controls (CT) [14]. The CJD dataset was included in this analysis because both AD and CJD are conformational disorders that share common clinical, neuropathological, and pathogenetic mechanisms [28,29]. Unlike typical AD, early onset AD exhibits atypical AD phenotypes that present high levels of total tau (T-tau) protein and/or positive 14-3-3 protein (p14-3-3) in the cerebrospinal fluid (CSF), reflecting intense neuronal degeneration similar to what is found in CJD [30]. GSE5281 (GPL570, HG-U133_Plus_2 Affymetrix Human Genome U133 Plus 2.0 Array) contains 161 samples, 74 of which are non-demented controls and 87 are affected with AD [15]. GSE36980 (GPL6244(HuGene-1_0-st) Affymetrix Human Gene 1.0 ST Array (transcript (gene) version) released Apr. 17, 2013) consists of microarray data from the gray matter of the frontal and temporal cortices and hippocampi derived from 80 postmortem brains of 33 AD patients and 47 controls [16]. GSE39420 (GPL11532: (HuGene-1_1-st) Affymetrix Human Gene 1.1 ST Array (transcript (gene) version) released on Jan. 20, 2015) consists of microarray data from 14 AD patients and 7 controls [17]. The controls were cognitively normal healthy cohorts with no history of AD. No ethics committee approval or patient consent was required for the present study.

### 2.2. Identification of Differentially Expressed Genes (DEGs)

DEGs were identified using the LIMMA package of R [31]. The Benjamini–Hochberg correction method was used for the *p*-value adjustment of the false discovery rate (FDR). An FDR of < 0.05 and |log[fold change (FC)]| cut off point were set for DEG selection. The Multiple List Comparator (https://www.molbiotools.com/listcompare.html, accessed on 21 September 2021), a web tool, was used to visualize the intersecting DEGs and to generate a Venn diagram to visualize overlapping DEGs. These overlapping DEGs were used for further bioinformatics analysis to uncover the molecular mechanism of AD.

### 2.3. Brain-Specific Gene Co-Expression, Protein–Protein Interaction (PPI) Networks, and Gene-Set-Enrichment Analysis (GSEA) of DEGs

We explored the NetworkAnalyst server (https://www.networkanalyst.ca/, accessed on 21 September 2021) [32] to conduct a brain-specific gene co-expression analysis. In accordance with the protocol described in previous studies [33,34], the DEGs were uploaded to the GENELIST module of the server and were analyzed for specific selected parameters: organism (*Homo sapiens*), ID type (gene symbol), tissue-specific co-expression (brain) analysis minimum-order network analysis. The Kyoto Encyclopedia of Genes and Genomes (KEGG) pathways and gene ontology (GO) enrichment analyses of the DEGs were conducted using the Enrich server [35,36]. The official gene symbols of the DEGs were uploaded into the Enrich server and were analyzed for the KEGG and GO terms under the default enrichment cutoff value of p < 0.05. The search tool for retrieval of interacting genes/proteins (STRING) server (http://string-db.org/, accessed on 21 September 2021, v10.5) [37] was used to construct the PPI network of the DEGs. The Entrez gene symbols of the DEGs were uploaded to the multiple protein modules of the STRING server and were analyzed for known and predicted PPI interactions in Homo sapiens under the high confidence (0.70) search and at a significant level of *p* < 0.05.

### 2.4. Analysis of Gene Disease-Specific Associations of the DEGs

We analyzed the disease-specific associations of DEGs by exploring the disease/phenotype-specific filters of the OPENTARGET platform (https://www.targetvalidation.org/, accessed on 21 September 2021) at a search score of 0.15. The OPENTARGET platform is a bio-web algorithm that integrates genetic, omic, and chemical data to identify the involvement of genes in diseases and aids in systematic drug target identification and prioritization [38].

### 2.5. MicroRNA (miRNA) Regulatory Network Analysis of the DEGs

The miRNA regulatory targets of the DEGs were collected from experimentally verified databases (TarBase, mir2disease, and miRTarBase) and predicted databases (miRanda and targetscan). The miRNA regulatory network was visualized using the visNetwork R packages. In addition, we used the miRNA Enrichment Analysis and Annotation (miEAA) tool (https://ccb-compute2.cs.uni-saarland.de/mieaa2/, accessed on 21 September 2021) to conduct a functional enrichment analysis of the miRNA targets [39]. Analysis was conducted using FDR (Benjamini–Hochberg) a *p*-value adjustment of 0.05 and a minimum required hit of four miR.

### 2.6. In Silico Evaluation of the Drug-Likeness, Pharmacokinetics (PKs), Blood–Brain Barrier (BBB) Permeability and Acute Rat Toxicity Study of Antrocin

We analyzed the drug-likeness, pharmacokinetics (PKs), medicinal chemistry, and toxicity of antrocin using SwissADME software (http://www.swissadme.ch, accessed on 21 September 2021) [40] and computer-aided Prediction of Biological Activity Spectra (PASS) web resources (http://way2drug.com/dr, accessed on 21 September 2021) [41]. We used the blood–brain barrier (BBB) prediction server (https://www.cbligand.org/BBB/, accessed on 21 September 2021), which operates based on support vector machine (SVM) and LiCABEDS algorithms on four types of fingerprints from 1593 reported compounds [42] to analyze the BBB-permeation ability of antrocin. The permeation threshold of the server is 0.02. In addition, we also used the brain or intestinal estimated permeation method (BOILED-Egg) model [43] to further analyze the brain- and intestinal-permeation abilities of the compound based on its lipophilicity and polarity. The antrocin SMILES format was also uploaded to the SwissADME server and was analysed for the presence of pan-assay interference compound (PAINS) substructures [44]. The GUSAR software for quantitative structure-activity relationship (QSAR)/quantitative structure-property relationship (QSPR) modelling was used for the in silico prediction of the 50% lethal dose (LD50) values of antrocin for rats through four administration routes (intravenous (i.v.), intraperitoneal (i.p.), oral inhalation, and subcutaneous (s.c.)) [45]. The GUSAR software was developed based on training datasets from the SYMYX MDL Toxicity Database and consisted of approximately 104 chemical structures with data on acute rat toxicity represented by LD50 values (log10 (mmol/kg)).

### 2.7. Molecular Docking Studies

The three-dimensional (3D) structures of CAMK4 (PDB:2W4O), ATP6V1A (PDB:6XBY), TIPRL (PDB:5WOW), BNIP3 (PDB:2J5D), and TOMM70 (PDB:3FP3) were downloaded from the protein data bank (PDB). The mol2 file for the 3D structure of antrocin was obtained using the Avogadro molecular builder and visualization tool vers. 1.XX [46] before subsequently being transformed into PDB format using the PyMOL Molecular Graphics System, vers. 1.2r3pre. The PDB files of the crystal structures of the targets were transformed to pdbqt format using AutoDock Vina (vers. 0.8, Scripps Research Institute, La Jolla, CA, USA) [47]. Prior to molecular docking, the receptors were charged, hydrogen atoms were added, and water (H_2_O) molecules were removed [48]. Docking experiments were performed with AutoDock Vina (vers. 0.8) using default settings at a docking exhaustiveness of 8 with all of the bonds in the ligand rotated freely while considering the receptors to be rigid. A grid box of 40 × 40 × 40 Å in the X, Y, and Z dimensions and a spacing of 1.0 Å were used [49]. The docked complex was visualized and analyzed using the Discovery Studio visualizer vers. 19.1.0.18287 (BIOVIA, San Diego, CA, USA) [50] and the protein–ligand interaction profiler (https://plip-tool.biotec.tu-dresden.de/plip-web/plip/index, accessed on 21 September 2021) [51].

## 3. Results

### 3.1. Deregulated Expressions of ATP6V1A, BNIP3, CAMK4, TIPRL, and TOMM70 Associated with the Pathology of Neurodegenerative Dementia

**Figure 2:**
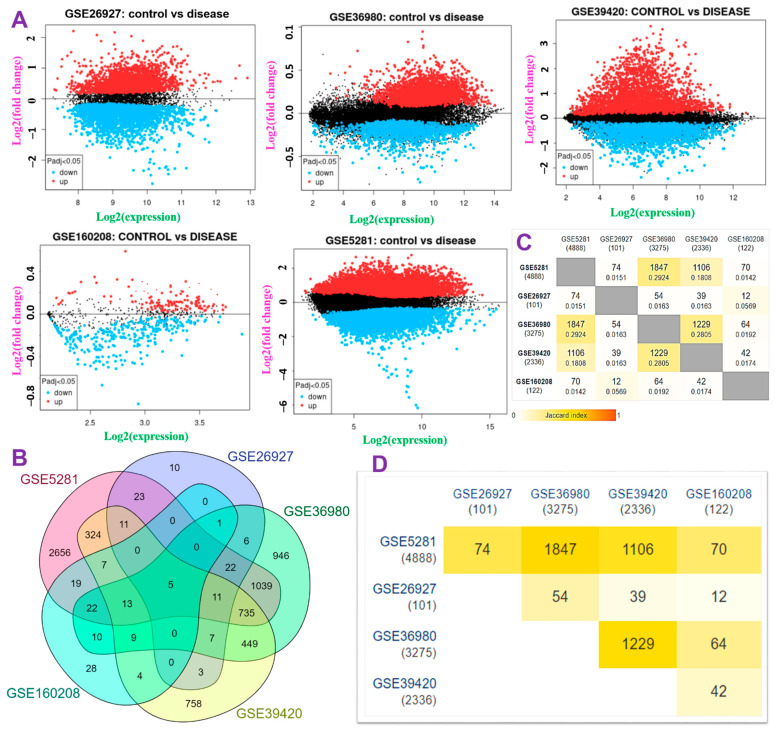
Identification of differentially expressed genes (DEGs) among patients with Alzheimer’s disease (AD) and healthy controls. (**A**) Volcano plot showing distributions of DEGs among AD patients and healthy controls. The top DEGs satisfied the criteria of log(multiple of change (MC) value) and *p* < 0.05. (**B**) Venn diagram of the DEGs across the six datasets. (**C**,**D**) Heatmap showing the Jaccard index of the DEGs in each of the datasets.

The flow chart of the study design for the in silico identification of the potential targets for Alzheime’sr disease and antrocin as a therapeutic candidate is shown in Figure 1. To retrieve the genes whose expression levels were significantly altered in AD brains compared to non-AD brains, we analyzed the transcriptomic data of multiple cohorts from microarray datasets (Table 1). The DEG distributions for each dataset are shown in a volcano plot (Figure 2A). The overexpressed DEGs were identified from each of the datasets and were based on an adjusted *p* < 0.05 when comparing the AD patients and the control subjects from each dataset (Figure 2B−D). Furthermore, we integrated the overexpressed DEGs from each dataset to identify overlapping and the most implicated DEGs in AD pathology. Five significantly overexpressed DEGs were identified: ATPase H+ transporting V1 subunit A (*ATP6V1A*), BCL2 interacting protein 3 (*BNIP3*), calmodulin-dependent protein kinase IV (*CAMK4*), TOR signaling pathway regulator-like (TIPRL), and translocase of outer mitochondrial membrane 70 (*TOMM70*) (Figure 2C).

### 3.2. ATP6V1A, BNIP3, CAMK4, TIPRL, and TOMM70 Localization and Differential Expressions in Brain Regions

**Figure 3:**
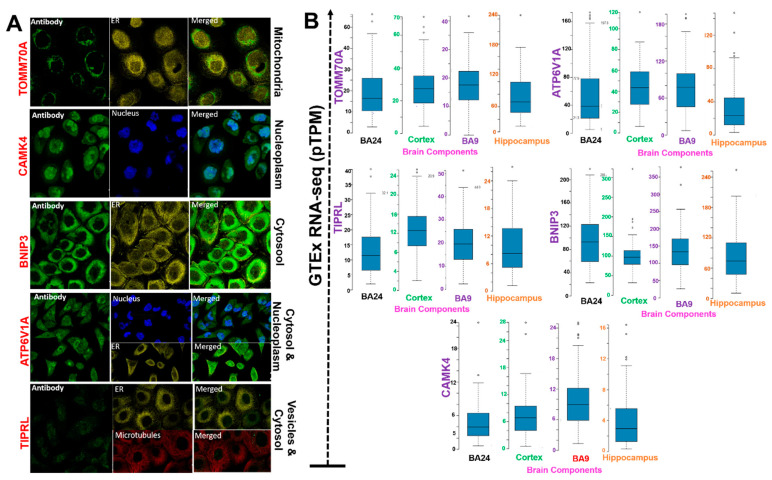
Localization and differential expressions of the ATP6V1A, BNIP3, CAMK4, TIPRL, and TOMM70 (**A**) immunofluorescence staining of the subcellular distribution of the proteins. Protein localization was detected based on the immunohistochemistry from the Human Protein Atlas (HPA) database. Scale bar = 20 μm (**B**) Bar plots showing differential expression levels of the genes in different brain regions.

To characterize the intracellular localization of the proteins, we acquired the indirect immunofluorescence data of distributions of the proteins within the nucleus, endoplasmic reticulum (ER), and microtubules of A-431 and A549 cells. We found that that *ATP6V1A*, *BNIP3*, *CAMK4*, *TIPRL*, and *TOMM70* were colocalized with the markers of different subcellular localizations: *ATP6V1A* (cytosol and nucleoplasm), *BNIP3* (cytosol), *CAMK4* (nucleoplasm), *TIPRL* (vesicles and cytosol), and *TOMM70* (mitochondria) (Figure 3A). In addition, we examined the expression levels of the DEGs in different regions of the brain. Taking the mean relative expression levels of these genes in different brain regions into account, we found that the frontal cortex (BA9) exhibited significantly (*p* < 0.05) higher DEG expression levels. In contrast, the hippocampus exhibited the lowest *ATP6V1A*, *BNIP3*, *CAMK4*, *TIPRL*, and *TOMM70* expression levels compared to the other parts of the brain that were analyzed (Figure 3B, Table 2).

### 3.3. MicroRNA (miR) Regulatory Network and Brain-Specific Gene Interactions of ATP6V1A, BNIP3, CAMK4, TIPRL, and TOMM70

**Figure 4:**
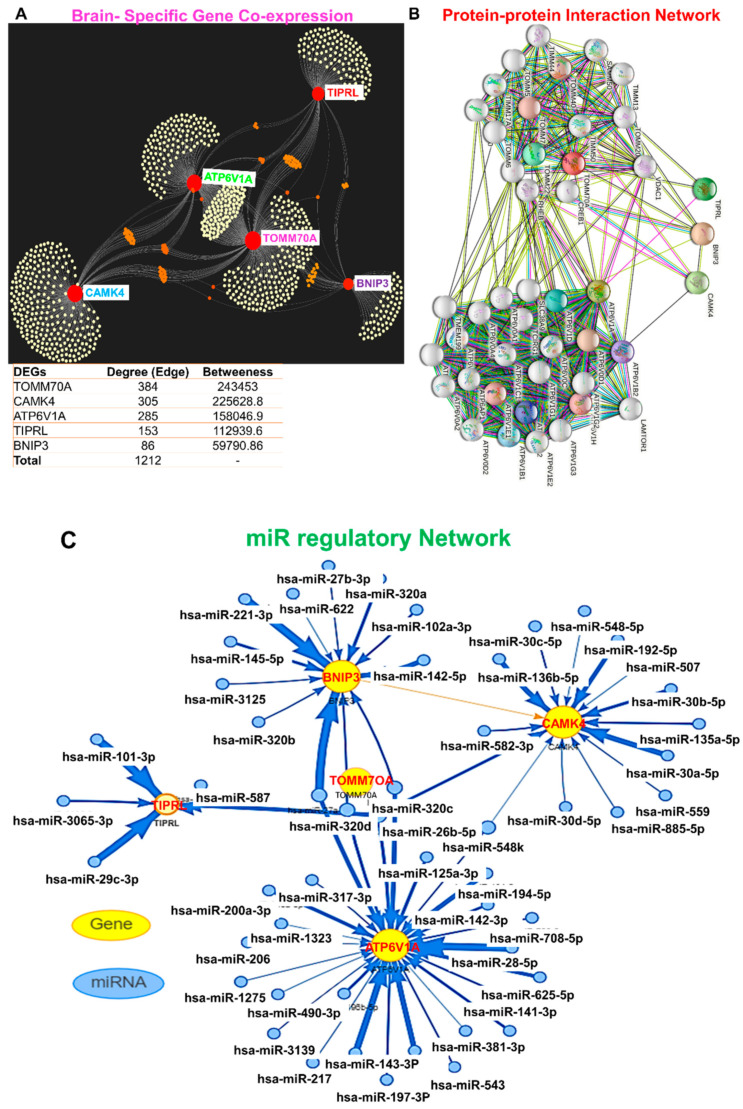
MicroRNA (miR) regulatory network and brain-specific gene interactions of *ATP6V1A*, *BNIP3*, *CAMK4*, *TIPRL*, and *TOMM70* (**A**) Network plot of brain-specific gene co-expressions of the gene set, (**B**) protein–protein interaction (PPI) network, and (**C**) miR regulatory networks of target genes.

**Figure 5:**
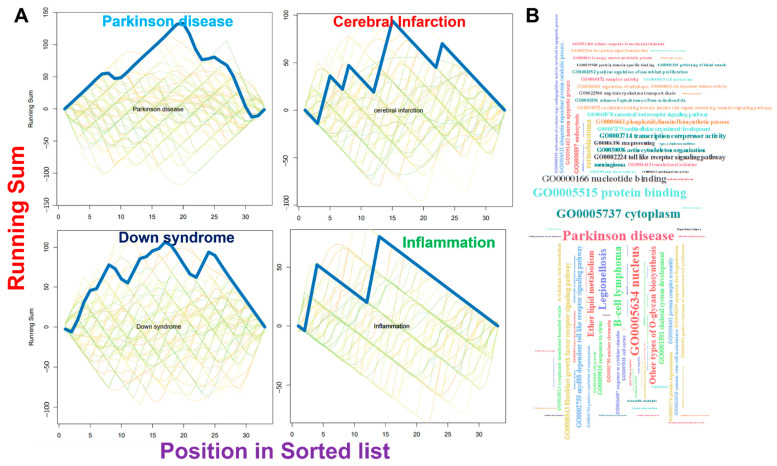
MicroRNA (miR) regulatory network enrichment of the DEGs. (**A**) Specific diseases enrichment plot and (**B**) cloud plot of the total enrichment of the DEGs.

We conducted a tissue-specific gene co-expression analysis and found that each of the DEGs exhibited brain-specific gene co-expression clustering with 986 clustering nodes and 1212 clustering edges (Figure 4A). Specifically, we observed higher clustering co-expression with optic atrophy 1 (OPA1), integrin alpha FG-GAP repeat containing 1 (ITFG1), 3-oxoacid CoA-transferase 1 (*OXCT1*), ATPase, Ca++ transporting, cardiac muscle, slow twitch 2 (*ATP2A2*), *MAPK1*, cyclin dependent kinase 14 (*CDK14*), *MAP2K4*, tyrosine 3-monooxygenase/tryptophan 5-monooxygenase activation protein beta (*YWHAB*), parkin RBR E3 ubiquitin protein ligase (*PARK2*), cytidine monophosphate N-acetylneuraminic acid synthetase (*CMAS*), heat shock protein family A member 12A (*HSPA12A*), and regulator of G protein signaling 17 (*RGS17*). Furthermore, the PPI interaction network yielded 45 nodes and 435 edges, with an average local clustering coefficient of 0.813 and PPI enrichment *p*-value of <1.0 × 10^−16^. The PPI network revealed that TOMM70A and ATP6V1A exhibited very high protein family-specific clustering interactions while BNIP3, CAMK4, and TIPRL formed a very minimal cluster (Figure 4B). We queried the miRNA targets of the DEGs to gain further insight into their pathological role. We found that DEGs are targeted by several miR-regulatory networks (Figure 4C) that are significantly associated with the pathogenesis of several CNS diseases including Parkinson’s disease, Down syndrome, inflammation, cerebral infarction, and several other diseases (Figure 5).

### 3.4. ATP6V1A/BNIP3 and CAMK4/TIPRL/TOMM70 Are Associated with Mitochondrial Dysfunction, Inflammatory Processes, and Various Pathways Involved in AD Pathogenesis

**Figure 6:**
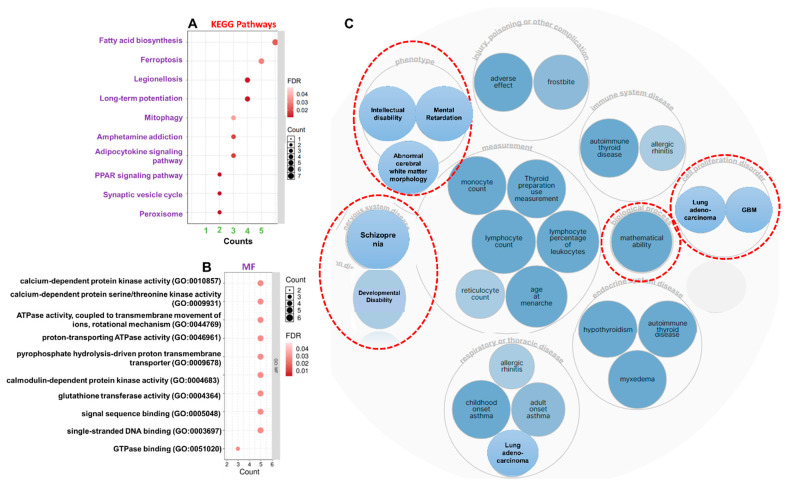
ATP6V1A/BNIP3 and CAMK4/TIPRL/TOMM70 are associated with several pathways and processes associated with brain functioning and disabilities. (**A**) KEGG pathways and (**B**) molecular function enrichment of ATP6V1A/BNIP3 and CAMK4/TIPRL/TOMM70. (**C**) Bubble plot showing the gene–disease association network of the gene set. GBM; glioblastoma multiforme.

To better understand the most common biological processes and pathways altered by dementia generation and progression, we analyzed disease associations, KEGG pathways, and the ontological enrichment of DEGs. Our results revealed the enrichment of fatty acid biosynthesis, ferroptosis, long-term potentiation, amphetamine addiction, adipocytokine signaling, peroxisome proliferator-activated receptor (PPAR) signaling, synaptic vesicle cycling, and peroxisome as major pathways associated with the DEGs (Figure 6A), while enriched molecular functions in the signatures were mainly associated with ATPase and calcium-dependent protein kinase activities (Figure 6B). In addition, biological processes, including the regulation of myeloid leukocyte differentiation, dendritic cell cytokine production, myeloid dendritic cell differentiation, cellular response to oxygen levels, and the mitochondrial protein catabolic process, were significantly enriched (Table 3). The gene–disease analysis further revealed that the ATP6V1A/BNIP3 and CAMK4/TIPRL/TOMM70 signatures were associated with several brain-related disorders, including developmental disabilities, schizophrenia, intellectual disabilities, abnormal cerebral white matter morphology, and mental retardation (Figure 6C). Collectively, the findings from this study suggest that ATP6V1A/BNIP3 and CAMK4/TIPRL/TOMM70 are associated with mitochondrial dysfunction, inflammatory processes, and various pathways involved in AD pathogenesis.

### 3.5. In Silico Pharmacokinetics, BBB Permeability and Acute Toxicity of Antrocin

**Figure 7:**
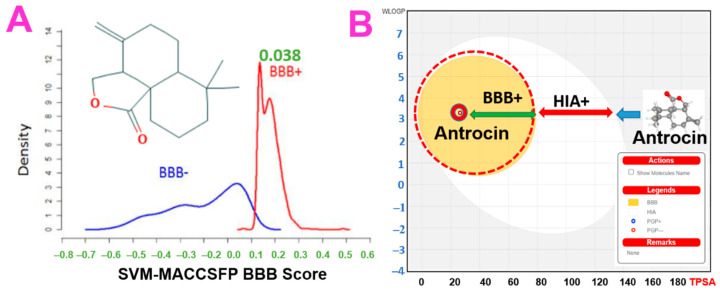
Two-dimensional (2D) structure and blood–brain barrier (BBB) permeation ability of antrocin. The (**A**) support vector machine (SVM)/LiCABEDS algorithms and (**B**) BOILED-Egg) model of BBB permeability of antrocin.

The preclinical evaluation of drug PKs can aid in the drug development process by providing a rationale for the selection of efficacious drug doses and treatment schedules [52]. The BBB is an important factor that limits the effectiveness of most chemotherapies against AD. Herein, we evaluated the BBB permeation ability (Figure 7) of antrocin, and our results revealed a BBB permeability score of 0.038 (Figure 7, Table 4) on a BBB permeant threshold of 0.02, suggesting that antrocin is BBB permeant and thus would be valuable for AD treatment. Our results also revealed good predictions for ADMET properties, drug-likeness, adherence to Lipinskís rules, no PAINS alerts, and that it is a non-inhibitor of CYP1A2, CYP2C19, CYP2D6 and CYP3A4 (Table 5). QSAR modeling of acute toxicity in rats revealed that antrocin had LD_50_ values of 618, 26, 804.3, 517.3 g/kg body weight for the i.p., i.v., o.p, and s.c. administrative routes, respectively (Table 4), suggesting that the compound has a high safety profile, especially when administered orally. In addition, antrocin demonstrated high environmental safety, as measured by the bioaccumulation factor, *Daphnia magna*, fathead minnow, and *Tetrahymena pyriformis*.

### 3.6. Molecular Docking Profiles of Antrocin with ATP6V1A, BNIP3, CAMK4, TIPRL, and TOMM70

Our molecular docking analysis revealed that antrocin exhibited strong interactions with the crystal structures of CAMK4 (PDB:2W4O), BNIP3 (PDB:2J5D), TIPRL (PDB:5WOW), TOMM70 (PDB:3FP3), and ATP6V1A (PDB:6XBY) and demonstrated the respective binding affinities of −6.70, −5.80, −6.60, −6.80, and −5.90 kcal/mol. Our analysis of interactions between the target gene and antrocin revealed that antrocin interacted with the gene targets through several hydrogen bonds, π-alkyl, van der Waals forces (Table 6), and several hydrophobic contacts (Table 7).

**Figure 8:**
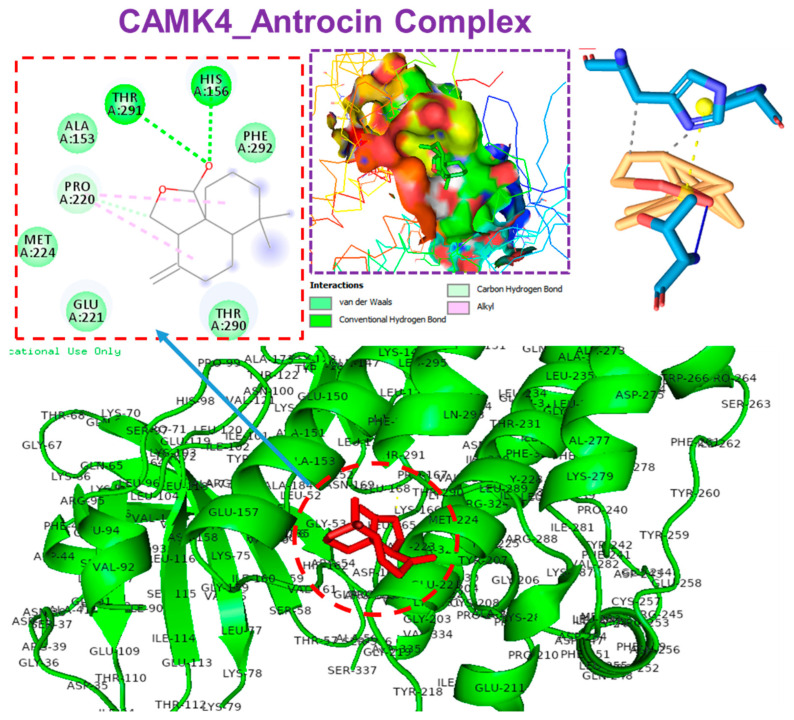
Molecular docking profile of CAMK4 with antrocin.

**Figure 9:**
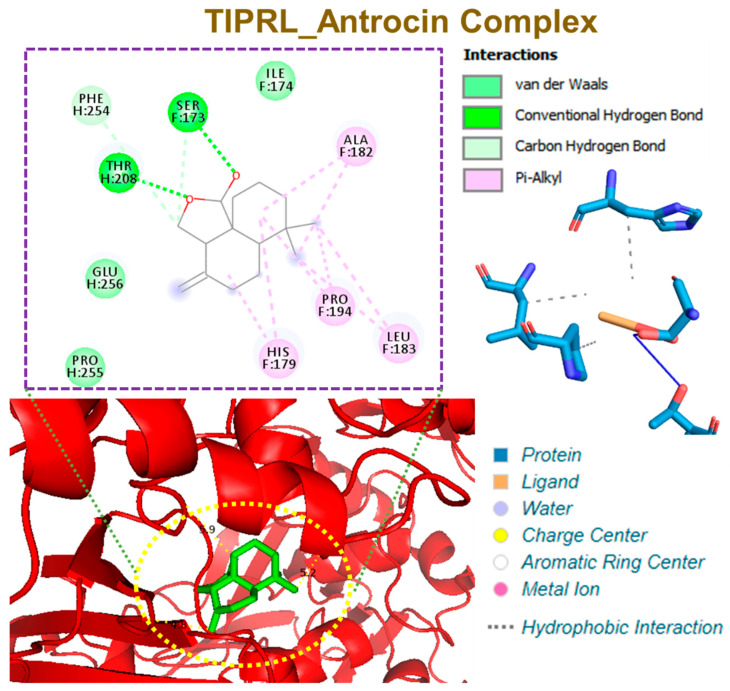
Molecular docking profile of TIPRL with antrocin.

Antrocin bound to CAMK4 with three hydrogen bonds, including THR291, HIS156, and PRO220, with the respective binding distances of 2.72, 1.92, and 3.65 Å. In addition, the antrocin–CAMK4 complex was further stabilized by several van der Waals forces that were formed around the backbone of antrocin with the amino acid residues of MET224, GLU221, THR290, PHE292, and ALA153 in the binding cavity of CAMK4. However, only two hydrophobic contacts (PRO220A and HIS156A) existed between antrocin and the binding cavity of CAMK4 (Figure 8). Antrocin was bound with TIPRL with three hydrogen bonds, including THR208, SER173, and PHE254, with the respective binding distances of 2.03, 2.97, and 3.78 Å, with several π-alkyl, van der Waals forces, and various hydrophobic contacts (Figure 9).

**Figure 10:**
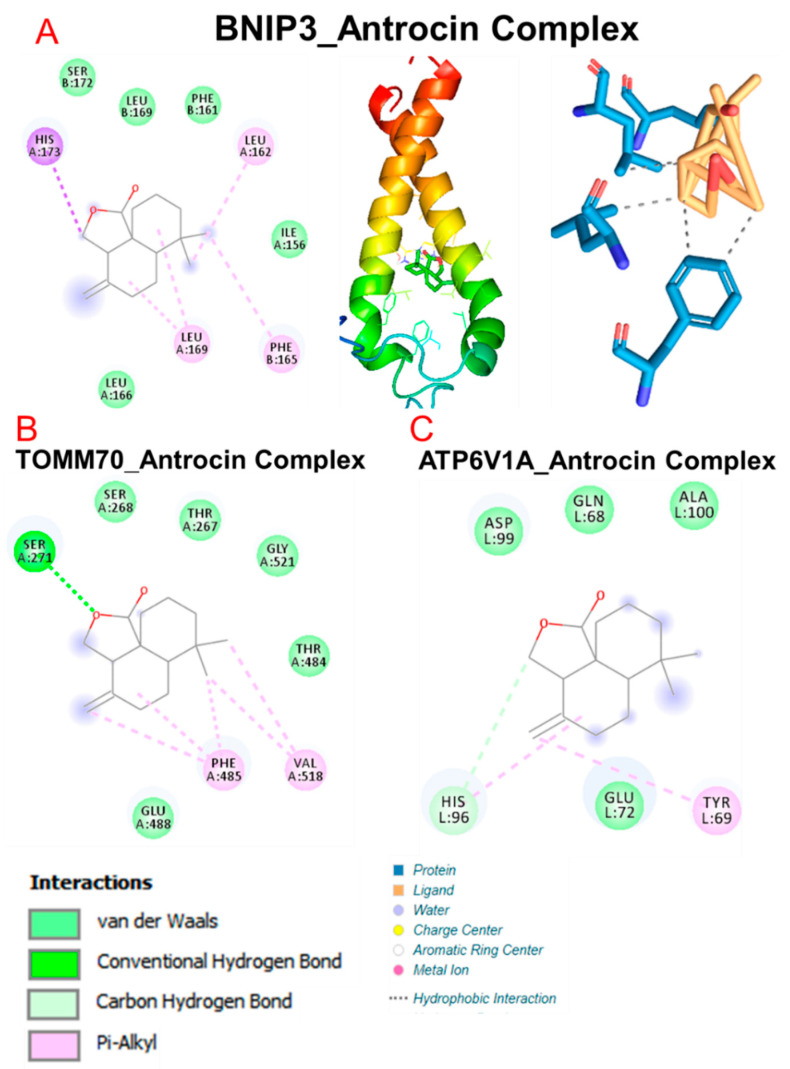
Molecular docking profiles of BNIP3, TOMM70, and ATP6V1A with antrocin. The two dimensional structure of the receptor-ligand complexes formed between antrocin and (**A**) BN1P3 (**B**) TOMM70, and (**C**) ATP6V1A.

No conventional hydrogen bonds were observed between antrocin and BNIP3, while only a single hydrogen bond was observed between the ligand (antrocin) and the receptors: TOMM70 (SER271, 2.40 Å) and ATP6V1A (HIS96, 2.16 Å). In addition, several van der Waals forces and hydrophobic contacts were also observed between antrocin and the receptors: BNIP3, TOMM70, and ATP6V1A (Figure 10).

## 4. Discussion

AD, one of the most common types of dementia, afflicts millions of people globally. In addition to its negative effects on physical and mental health, AD places a huge burden on both individuals and societies [53]. Although a number of medications are available for the relief of AD symptoms, no cure for AD presently exists [54]. Therefore, it is evident that identifying DEGs that play pivotal roles in AD pathogenesis is an important step towards the development of appropriate therapeutic strategies. A comparative analysis of the transcriptomic data between AD subjects and healthy controls revealed important molecular pathways and biological processes altered by AD generation and progression.

In terms of upregulated genes, our pathway enrichment results included fatty acid biosynthesis, ferroptosis, long-term potentiation, amphetamine addiction, adipocytokine signaling, PPAR signaling, synaptic vesicle cycle, and peroxisome, all of which are commonly accepted components of the pathogenesis and pathological changes of AD [55,56,57,58,59].

Inflammatory processes play fundamental roles in the pathogenesis of AD [60]. Activated cells strongly produce inflammatory mediators such as proinflammatory cytokines, chemokines, leukotrienes, reactive oxygen species, and other radicals [61,62]. In addition, the enrichment of biological processes, including the regulation of myeloid leukocyte differentiation, dendritic cell cytokine production, myeloid dendritic cell differentiation, cellular responses to oxygen levels, and the catabolism of mitochondrial proteins, indicate important factors, all of which are associated with activation of the inflammatory processes involved in the pathogenesis of AD [61,62].

Furthermore, the enrichment of the regulation of myeloid leukocyte differentiation, the positive regulation of dendritic cell cytokine production, cellular responses to oxygen levels, and the myeloid dendritic cell differentiation in the biological process ontologies of DEGs suggest a mitochondrial dysfunction event, which is an important factor in the pathogenesis of AD and other neurodegenerative disorders such as PD [63]. Collectively, the findings from this study suggest that ATP6V1A/BNIP3/CAMK4/TIPRL/TOMM70 are associated with mitochondrial dysfunction, inflammatory processes, and various pathways involved in AD pathogenesis.

ATP6V1A is a multi-subunit enzyme that is associated with synaptic vesicle proton gradient generation in the brain, energy metabolism, and ATP synthesis. In agreement with a previous study [64], our comprehensive analyses of DEGs, enrichment, and PPI networks strongly suggest that ATP6V1A may play an important role in AD pathogenesis. Moreover, ATP6V1A maturations were implicated in the onset of developmental encephalopathy with epilepsy, suggesting its role in regulating neuronal development [65]. A clinical study by Dutta et al. [66] reported that two patients with de novo mutations (T607I and I554F) in the C-terminus of the TOM70 protein exhibited white matter abnormalities, hypotonia, hyperreflexia, dystonia, and cognitive deficits.

Furthermore, our brain-specific gene co-expression analysis revealed that the DEGs exhibited gene co-expression clustering with *OPA1*, *ITFG1*, *OXCT1*, *ATP2A2*, *MAPK1*, *CDK14*, *MAP2K4*, *YWHAB*, *PARK2*, *CMAS*, *HSPA12A*, and *RGS17*, all of which were reported to play essential roles in the regulation of cell proliferation and AD pathogenesis. Similar to the results obtained in our analysis, Zhang et al. [64] detected the upregulation of *ATP6V1A* and OXCT1 in the AD cohorts compared to in the healthy cohorts [64]. Experimental evidence links *OPA1* to the pathogenesis of Parkinsonian syndrome and dementia [63], while *OXCT1* plays a central role in extrahepatic ketone body catabolism [67]. Collectively, the results of the present study suggest that *CAMK4*, *BNIP3*, *TIPRL*, *TOMM70*, and *ATP6V1A* are important DEGs that are associated with the pathogenesis of AD and thus serve as attractive targets in the development of therapeutic intervention.

Molecular docking is widely employed as an in silico model for elucidating the interactions between proteins and ligands and also in the estimation of the binding affinities of protein–ligand complexes [68,69]. This enables the depiction of the behavior of a drug candidate within the binding cavity of a receptor and ultimately gives an idea of the biological processes that could be modulated by the drug candidate [70,71]. Consequently, we conducted a molecular docking study to elucidate the potential druggability of the target genes by antrocin, a bioactive natural product. Interestingly, our molecular docking study suggested that antrocin exhibited strong interactions with the crystal structures of CAMK4 (PDB:2W4O), BNIP3 (PDB:2J5D), TIPRL (PDB:5WOW), TOMM70 (PDB:3FP3), and *ATP6V1A* (PDB:6XBY), with estimated binding affinities of −6.70, −5.80, −6.60, −6.80, and −5.90 kcal/mol, respectively. Our analysis of the protein–antrocin complexes revealed that antrocin interacted with the targets created by several hydrogen bonds, π-alkyl, van der Waals forces, and several hydrophobic contacts. Non-covalent interactions, such as hydrogen, hydrophobic and ionic bondings, and van der Waals forces, play crucial roles in stabilizing the interactions between drug candidates and their protein targets [72,73]. The higher number of interactions that antrocin has with CAMK4, TOMM70, and T1PRL could be responsible for the stronger affinities that antrocin has for these proteins than it does for BNIP3 or ATP6V1A. The van der Waals forces created around the antrocin backbone with the respective amino acids of the proteins would create a strong cohesive environment, which would further stabilize the complexes [74]. However, the lower affinity of antrocin for *BNIP3* could be attributed to the absence of H-bonding in the antrocin–*BNIP3* complex.

The BBB is a limiting factor in the therapeutic effects of most medications for treating AD. Although a number of AD drug treatment trials targeting BBB dynamics have been conducted, most of them have failed due to inadequate permeability. In addition, a subset of AD cases with chronic hypoperfusion features is complicated by an inadequate BBB [75]. Therefore, BBB permeation is an important factor that must be considered in the development of a new drug for AD. Interestingly, our in silico studies indicated that antrocin has high BBB permeability potential. Only a small fraction of small molecules cross the BBB via lipid-mediated free diffusion, and these molecules must have <8 hydrogen bonds and a molecular weight of < 400 Daltons (Da) [76]. Interestingly, antrocin satisfied these criteria, with a MW of 234.33 Da, a surface area of 26.30 Å², and forms two hydrogen bonds. The MW threshold is a property of all biological membranes [77]. In agreement with our findings, a screening of CNS-acting drugs showed that all brain drugs have an MW <426 Da [78]. The BBB permeability decreases 100-fold when the surface area of a drug is increased from 50 Å^2^ to 100 Å^2^ [78], which would be the case when the MW of a drug increases from 300 Da to 450 Da [76]. Antrocin demonstrated good predictions for ADMET properties, drug-likeness, and adherence to Lipinskís rules. The pan-assay interference compounds (PAINS) are known as substructures of promiscuous molecules that are responsible to yield false-positive biological activities [44]. No alert was evidenced for antrocin in the PAINS analysis, suggesting that this compound is non-promiscuous and hence has potential for use in target therapy [79]. Our results suggest that antrocin is a BBB permeant drug-like molecule and hence may have translational potential for treating AD and other brain disorders.

Intestinal absorption and brain penetration are important factors to consider for therapeutic agents that are intended to treat neurodegenerative diseases in response to oral exposure [80]. Our results showed that antrocin is predicted to be highly absorbed by the human gastrointestinal tract. Increasing evidence has revealed that that the p-glycoprotein (P-gp) efflux may interact with drugs and consequently prevent their absorption by actively repelling them into the lumen of the intestine [81]. Our results indicated that antrocin is not a p-gp substrate, and this could account for the high predicted GIT absorption of antrocin. In the case of metabolism, various cytochrome P450 isoenzymes were evaluated, showing that antrocin is not a substrate for metabolism by CYP1A2, CYP2C19, CYP2D6, and CYP3A4 isoenzymes. In addition to BBB permeability, the high bioavailability of antrocin (0.55, Table 5) could ultimately favour its accessibility to the brain. Altogether, our results suggest that the oral administration of antrocin is likely to result in safer bioavailability and access to the BBB, thus suggesting its potential for AD treatment.

Taken together, the present study strengthened our understanding of the molecular pathogenesis of AD, identified disease targets, and suggested the therapeutic potential of antrocin. The main limitation of our study is the lack of the direct confirmation of these genes by reverse-transcription polymerase chain reaction (RT-PCR). However, experimental validation and the detailed therapeutic efficacy of antrocin in an AD animal model is currently ongoing in our laboratory. In addition, the RT-quantitative (q)PCR validation of these identified targets in clinical samples is ongoing.

## 5. Conclusions

Our findings identified a potential new gene signature, ATP6V1A/BNIP3/CAMK4/ TIPRL/TOMM70, as critical molecular markers in mitochondrial dysfunction, inflammatory processes, and various pathways participating in AD parthenogenesis. Additionally, in silico assays and docking experiments revealed that antrocin interacted well with the crystal structures of the proteins and exhibited a low acute toxicity level based on the in silico modelling of the various administration routes. Our results suggest the potential of antrocin in targeting the DEGs; however, further investigation is warranted for the clinical translation of antrocin to ameliorate and prevent AD.

## Figures and Tables

**Table 1:** Characteristics of the subjects and the microarray datasets included in this study.

Datasets	Platform	No. of Cases	No. of Controls	Total No.	Mean Age (Years)
GSE5281	GPL570	87	74	161	79.8 0 ± 9.10
GSE160208	GPL29311	27	20	47	NA
GSE26927	GPL6255	63	56	119	63.65 ± 10.83
GSE36980	GPL6244	33	47	80	NA
GSE39420	GPL11532	14	7	21	55.66 ± 1.93

NA, not available.

**Table 2:** Mean expression data of the *ATP6V1A*, *BNIP3*, *CAMK4*, *TIPRL*, and *TOMM70* genes in different brain regions.

	GTEx RNA-seq (pTPM)				
Brain Region	TIPRL	TOMM70A	CAMK4	ATPV1A	BNIPS
Anterior cingulate cortex (BA24)	12.90 ± 7.90 ^b^	19.10 ± 11.70 ^b^	4.70 ± 3.30 ^a^	53.40 ± 42.60 ^b^	98.90 ± 45.70 ^b^
Cortex (central)	12.30 ± 4.50 ^b^	19.20 ± 7.20 ^b^	7.50 ± 4.00 ^b^	43.81 ± 20.00 ^b^	98.63 ± 33.20 ^b^
Frontal cortex (BA9)	19.90 ± 9.80 ^c^	27.80 ± 13.10 ^c^	9.30 ± 5.20 ^b^	77.60 ± 42.50 ^b^	136.72 ± 55.03 ^b^
Hippocampus	9.70 ± 5.70 ^a^	14.00 ± 8.20 ^a^	4.00 ± 3.30 ^a^	31.70 ± 27.50 ^a^	80.43 ± 45.05 ^a^

Raw data were downloaded from Human Protein Atlas (HPA) repositories. Data were analyzed, and results are presented as the mean ± standard error of the mean. Values with different superscript letters in a column significantly differ (a > b > c) at *p* < 0.05. BA24; Brodmann area 24, BA9; Brodmann area 9.

**Table 3:** Biological processes enriched by the target genes.

Index	Name	*p*-Value	Adjusted *p*-Value	Odds Ratio	Combined Score
1	catabolism of mitochondrial proteins (GO:0035694)	0.003296	0.04581	399.68	2284.21
2	response to increased oxygen levels (GO:0036296)	0.003844	0.04581	333.05	1852.16
3	regulation of myeloid leukocyte differentiation (GO:0002761)	0.004392	0.04581	285.46	1549.44
4	negative regulation of mitochondrial fusion (GO:0010637)	0.004392	0.04581	285.46	1549.44
5	positive regulation of dendritic cell cytokine production (GO:0002732)	0.004392	0.04581	285.46	1549.44
6	mitochondrial fragmentation involved in apoptotic process (GO:0043653)	0.004940	0.04581	249.76	1326.33
7	negative regulation of membrane potential (GO:0045837)	0.004940	0.04581	249.76	1326.33
8	positive regulation of mitochondrial membrane permeability involved in apoptotic process (GO:1902110)	0.004940	0.04581	249.76	1326.33
9	cellular response to oxygen levels (GO:0071453)	0.005488	0.04581	222.00	1155.57
10	myeloid dendritic cell differentiation (GO:0043011)	0.005488	0.04581	222.00	1155.57

**Table 4:** In silico acute rodent toxicity assays for antrocin.

	**LD_50_**		
Administration Route	Log10(mmol/kg)	mg/kg	OECD classification
Intraperitoneal (i.p)	0.421	618	Class 5
Intravenous (i.v)	−0.955	26	Class 3
Oral gavage (o.p)	1.536	804.3	Non-toxic
Subcutaneous (s.c)	0.344	517.3	Class 4
**Environmental Toxicity**			
Bioaccumulation factor Log10(BCF)			1.521
Daphnia magna LC_50_-Log10(mol/L)			4.594
Fathead Minnow LC_50_ Log10(mmol/L)			−1.648
*Tetrahymena pyriformis* IGC_50_-Log10(mol/L)			0.856

The toxicity classification was based on the acute rodent toxicity chemical classification by the OECD Project. LD_50_, 50% lethal dose.

**Table 5:** Drug-like and properties of antrocin.

Properties	Antrocin	Reference Value
Formula	C_15_H_22_O_2_	−
M.W(g/mol)	234.33	150−500
R-bonds	0	0−9
H-bond ACC.	2	0−10
H-bond DON.	0	0−5
Molar Refractivity	68.17	40 ~ 130
TPSA (Å²)	26.30	20−130
Fraction Csp3	0.80	0.25 ~ < 1
Log Po/w (XLOGP3)	3.44	−0.7 ~ 5
Consensus Log Po/w	3.31	≤3.5
Drug-likeness (Lipinski rule)	Yes (0 violation)	MLOGP ≤ 4.15, M.W ≤ 500, H-bond ACC ≤ 10, H-bond DON ≤ 5
Bioavailability Score	0.55	>0.1 (10%)
BBB-permeation (SVM_MACCSFP)	0.038	≥0.02
Synthetic accessibility	4.18	1 (very easy) to 10 (very difficult).
PAINS	0 alert	No alert
P-gp substrate	No	
CYP1A2 inhibitor	No	
CYP2C19 inhibitor	No	
CYP2D6 inhibitor	No	
CYP3A4 inhibitor	No	
Log *K*_p_ (skin permeation)	−5.29 cm/s	<−3.5

R-bond; Num. rotatable bonds; H-bond ACC; Num. H-bond acceptors, H-bond DON; H-bond donors, TPSA; topological polar surface area, BBB; blood–brain barrier, P-gp; P-glycoprotein. Kp; permeability coefficients (kp).

**Table 6:** Molecular docking profiles between antrocin and various targets.

Interaction	CAMK4	BNIP3	TIPRL	TOMM70	ATP6V1A
ΔG = (kcal/mol)	−6.70	5.80	−6.60	−6.80	−5.90
Conventional H-bonds	THR291 (2.72 Å) HIS156 (1.92 Å) PRO220 (3.65 Å)		THR208 (2.03 Å) SER173 (2.97 Å) PHE254 (3.78 Å)	SER271 (2.40 Å)	HIS96 (2.16 Å)
π-alkyl	PRO220	LEU169 PHE165 LEU162	ALA182 PRO194 LEU183 HIS179	PHE485 VAL518	TYR69
π-sigma		HIS173			
van der Waals forces	MET224 GLU221 THR290 PHE292 ALA153	SER172 LEU169 PHE161 ILE156 LEU166	PHE254 GLU256 PRO255	SER268 THR267 GLY521 THR484 GLU488	ASP99 GLN68 ALA100 GLU72

**Table 7:** Hydrophobic contact between antrocin and various targets.

Target	Amino Acid Residue	Ligand Atom	Protein Atom	Distance (Å)
**BNIP3**	PHE165B	868	628	3.69
	PHE165B	863	629	3.66
	LEU166A	873	207	3.59
	LEU169A	869	231	3.35
	LEU169A	872	230	3.85
	LEU169B	868	661	3.40
**TIPRL**	HIS179F	42301	19984	3.68
	LEU183F	42299	20029	3.63
	PRO194F	42295	20139	3.72
**TOMM70**	THR484A	5020	3672	3.69
	PHE485A	5014	3684	3.62
	PHE485A	5013	3686	3.51
	PHE485A	5020	3685	3.66
	VAL518A	5020	4029	3.53
**ATP6V1A**	TYR69L	17251	3978	3.69
	GLU72L	17251	4001	3.58
	HIS96L	17252	4191	3.64
**CAMK4**	HIS156A	2587	1013	3.90
	PRO220A	2588	2588	3.70

## Data Availability

The datasets generated and analyzed in this study can be made available upon reasonable request.
